# Integration of motion information in illusory motion perceived in stationary patterns

**DOI:** 10.1038/s41598-023-48265-4

**Published:** 2023-11-30

**Authors:** Taisuke Kobayashi, Eiji Watanabe

**Affiliations:** 1 Laboratory of Neurophysiology, National Institute for Basic Biology, Higashiyama 5-1, Myodaiji-Cho, Okazaki, Aichi 444-8787 Japan; 2 Department of Basic Biology, The Graduate University for Advanced Studies (SOKENDAI), Miura, Kanagawa 240-0193 Japan

**Keywords:** Visual system, Human behaviour, Computer modelling, Numerical simulations

## Abstract

Illusory motion is a phenomenon in which stationary images with repeating luminance gradient patterns appear to be moving. In this study, we conducted experiments focusing on illusory motion to verify the hypothetical rule that velocity information, extracted from local luminance patterns, is integrated by summation in visual information processing. This rule is based on the hypothesis of velocity integration, and could estimate perceived velocity of stimulus. The summation rule was evaluated by a psychophysical experiment. Our results showed that the summation rule unbiasedly predicted perceived velocity, suggesting that an algorithm for integrating velocity information in illusory motion is based on the summation rule. These results would contribute to understanding of the spatial integration of local motion signals in visual information processing.

## Introduction

A repeating pattern of luminance gradients can often make a pattern appear to be moving even though it is stationary. This phenomenon, referred to here as illusory motion, was first reported by Fraser and Wilcox in 1979^1^, and several variants have since been developed^2–5^. One of major focuses of illusory motion research has been on how motion signals are induced from luminance patterns in visual information processing^6–12^. Information processing for illusory motion is thought to reflect that for general motion perception. In this mechanism, motion is perceived by integrating local motion information in higher visual cortex detected in primary visual cortex^2^. However, spatial integration of local motion signals remains unclear.

Formulation of perceived velocity for illusory motion has been approached primarily through psychophysical studies. Atala-Gerard and Bach focused on discrete luminance gradient of black, gray 1, white, and gray 2 in "Rotating snakes" illusion designed by Akiyoshi Kitaoka^5^. They conducted psychological experiments to determine magnitude of illusory motion when luminance of gray 1 and gray 2 was varied. As a result, direction of motion varied based on the combination of luminance between gray 1 and gray 2^13^. Atala-Gerard and Bach used experimental results to test four hypotheses^6–8,10^. First, Conway et al. (2005) proposed a hypothesis that focused on differences in neural responses. They explained that local velocity information is detected from adjacent luminance pairs, and its integration leads to the illusory motion. Second, Backus & Oruc (2005) also proposed a hypothesis based on the fact of differential neural responses^14^. Perceptual velocity is calculated by adapting transfer function according to this neural response to the entire pattern and its temporal variations. Murakami et al. (2006) proposed a hypothesis suggesting that illusory motion occurs during eye movements due to processing of luminance distribution information by a nonlinear temporal filter. They further explained that local velocity information is detected from adjacent luminance pairs, and this motion is then perceived by integrating these pairs. Finally, Fermüller et al. (2010) also computed perceptual velocity from a combination of eye movements and a nonlinear temporal filter, using this filter for entire pattern. (However, as a result of validation, it was determined that hypothesis of Fermüller et al. was unable to replicate initial results).

Finally, it was found that these hypotheses failed to reproduce results of psychological experiments, indicating a need for hypothesis adjustments^15^. Bach and Atala-Gerard then introduced a novel mathematical framework to reproduce observed data^12^. In this hypothesis, perceived velocity is determined by integrating local velocities at each point derived from adjacent luminance values through a nonlinear filter. Therefore, we designed a validation experiment focusing on the hypothesis that the integration of local motion velocity is performed by a simple procedure such as linear summation. Note that, although the summation rule tested here calculates local motion velocity by summation, it is not inconsistent with the hypothesis suggesting that local motion velocity arises from the spatial average of information, as also mentioned by Bach and Atala-Gerard^12^. We first tested this hypothesis with a neural network model^16^ developed using predictive coding, which reproduces human perception as a computational principle, and obtained results that support this hypothesis^17^. In this study, we tested this local velocity summation theory in psychological experiments, utilizing patterns with discrete luminance gradients in color and grayscale.

**Figure 1:**
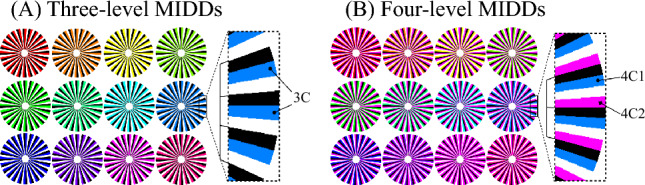
Motion Illusion-like Discrete Designs (MIDDs). Twenty-four visual stimuli (12 three-color MIDDs and 12 four-color MIDDs) used in the psychophysical experiment. Three-level MIDDs consist of three luminance levels: black, intermediate luminance level (3C), white; four-level MIDDs consist of black, intermediate luminance level 1 (4C1), white and intermediate luminance level 2 (4C2).

If the local velocity summation hypothesis is correct, it suggests that amount of illusory motion for stimuli with a more complex structure can be predicted from that for stimuli with a simple structure. We then tested whether amount of illusory motion could be predicted using two types of stimuli. In the experiments, motion illusion-like discrete design (MIDD) was prepared, as shown in Fig. 1 (see “Methods” section for details). Patterns with discrete luminance gradients, typified by Prof. Akiyoshi Kitaoka's "Rotating snakes" illusion^5^, are known to produce greater motion perception than smooth luminance gradients originally proposed^4^. At least three different luminance levels are required to create an asymmetric luminance gradient for perception of illusory motion. Therefore, the MIDD with three luminance levels is the stimulus with the most basic luminance gradient. If perceived velocity is sum of local velocities, and perceived velocities of three-level MIDDs are known, it should be possible to predict perceived velocity of four-level MIDD composed of combined three-level pattern. In these experiments, perceived velocities of three- and four-level MIDD were obtained to validate the summation rule for the illusory motion as described below. It has also been reported that color stimuli produce a greater amount of illusory motion compared to grayscale stimuli^6^. This suggests that color stimulus may elicit a mechanism distinct from that of luminance. In this experiment, we prepared both grayscale and color stimuli to examine effect of color on illusory motion.

In the following section, we first explain the summation rule for illusory motion tested in this paper. Then results of the psychophysical experiment are presented.

## Methods

### Motion illusion-like discrete designs (MIDDs)

Motion illusion-like discrete design (MIDD) used in these experiments is a disk with three or four luminance levels arranged in a repeated circular pattern (Fig. 1). Stimuli are based on those used in the experiments of Hisakata et al.^18^ and the optimized Fraser-Wilcox illusion^4^. A three-level MIDD consists of units with black, mid-luminance (3C), and white elements in sequence; a four-level MIDD consists of black, mid-luminance 1 (4C1), white, and mid-luminance 2 (4C2) in sequence. MIDD is configured with twenty-four of these units, arranged repetitively in a circular pattern.

In this study, we primarily tested the local velocity summation hypothesis, while also examining effect of color on perceived velocity. For this reason, both grayscale and color stimuli were used in the experiments. First, we prepared color MIDD images. Intermediate luminances (3C, 4C1, 4C2) were set using HLS color code, with Lightness and Saturation set to 0.5 and 1, respectively. Twelve hues were used by varying Hue from 0 to 330 degrees in 30 degrees increment. Displayed RGB values have been adjusted based on display calibration as described below. Luminance values correspond to measurements taken using these calibrated RGB values (see Supplementary Fig. S2). In addition to original color images, we prepared grayscale images that closely matched the measured luminance values of the respective color images. In total, 24 stimuli were prepared for three-level MIDDs. Twenty-four stimuli were actually used with hue of 4C2 fixed at 300 and hue of 4C1 was varied to reduce learning effect and heavy load on subjects.

To set outer diameter to 7 degrees, outer diameter of circular pattern of stimuli displayed on the screen was set to 382 pixels and inner diameter to 55 pixels.

### Psychophysical experiment

A psychophysical experiment was designed and implemented using Python program^19^ with OpenGL, based on the methods by Murakami et al.^8^, Hisakata et al.^18^ and Kobayashi et al.^20^. In this experiment, subjects were instructed to respond via keyboard whether each stimulus was rotated clockwise (CW) or counterclockwise (CCW). To quantify amount of illusory motion, we intentionally rotated stimuli and identified the condition under which illusory motion was canceled. We defined the cancellation velocity as equal to amount of illusory motion. To obtain response statistics, each condition was presented multiple times by randomly varying stimulus type and rotational velocity. Response data were collected and cancellation velocity was obtained by a constant method.

Subjects' faces were fixed at a distance of 50 cm from the screen and only right eye was used for observation. A stimulus with an outer diameter of 7 degrees and an inner diameter of 1 degree was presented for 0.5 s, positioned 12 degrees to left of center. Stimuli were luminance-calibrated using measured luminance characteristics of the display. Subjects viewed stimuli in peripheral vision by looking at a fixation point. After subject’s response, next stimulus was presented with a minimum interval of 1 s from previous stimulus. In informal observation, it was confirmed that amount of illusory motion was smaller for three-level stimuli compared to four-level stimuli. Therefore, the three-level experiment was designed with a more limited velocity range and increased presentation frequency compared to the four-level experiment. Finally, intended rotational velocities of stimulus were set to − 2.4 to + 2.4°/s and − 5.0 to + 5.0°/s, and intervals were set to 0.4°/s and 1.0°/s, for three- and four-level MIDDs, respectively. Each condition was presented 24 and 20 times respectively, resulting in total presentations of 624 and 440 times for each stimulus respectively.

Two types of stimuli were prepared: original images and these mirror-reversed version. This allowed us to control for bias in perceived rotational velocity. From statistical data obtained in this procedure, we calculated rotational velocity of stimulus when probability of responding that stimulus was rotated in CW and CCW directions was equal, using the probit analysis. Supplementary Fig. S1 shows the psychological curve obtained in the experiment. Perceived velocity of stimulus was then calculated as (cancellation velocity of reversed stimulus) − (cancellation velocity of original stimulus)/2.

In the psychophysical experiment, stimuli were displayed on an LCD monitor (ASUS, PB287Q). However, the relationship between input RGB levels and displayed luminance is not linear. Therefore, input RGB levels were corrected to show the intended color information prior to the psychophysical experiment.

First, we measured luminance by 256 shades of input RGB in increments of eight levels (ColorCAL MKII, Cambridge Research Systems). In addition, the luminance of white (255,255,255) and black (0,0,0) was measured. All measured luminances were normalized to white luminance after subtracting the offset caused by black luminance. Relationship between each displayed RGB (not input RGB) and its luminance, ${Y}_{R}$, ${Y}_{G}$, and ${Y}_{B}$ should exhibit linearity as,

$${\text{Y}}_{{\text{R}}} = \frac{{{\text{Y}}_{{{\text{R}}_{{{\text{Max}}}} }} }}{255}{\text{R}},\;{\text{Y}}_{{\text{G}}} = \frac{{{\text{Y}}_{{{\text{G}}_{{{\text{Max}}}} }} }}{255}{\text{G}},\;{\text{Y}}_{{\text{B}}} = \frac{{{\text{Y}}_{{{\text{B}}_{{{\text{Max}}}} }} }}{255}{\text{B}}$$

where ${{Y}_{R}}_{Max}$, ${{Y}_{G}}_{Max}$, and ${{Y}_{B}}_{Max}$ are luminance levels of R, G, and B at their maximum levels, respectively. Luminance is normalized, and as a result, it has no units. By determining ${Y}_{R}$, ${Y}_{G}$, and ${Y}_{B}$ from the displayed RGB values, it is possible to calculate the corrected input RGB from the measured levels. Luminance of each hue of HLS color space, displayed using corrected input RGB obtained by this procedure, is shown in Supplementary Fig. S2.

To use grayscale stimuli in the experiment, we measured luminance by 256 shades of grayscale input RGB in increments of eight levels. This calibration also involved pre-processing through offsetting and normalization, as previously described. Intermediate luminance of each grayscale stimulus corresponds to displayed RGB level that is closest to luminance for each hue after the calibration.

The five subjects were the authors, T.K. and E.W., and three other naive subjects, with normal (or corrected-to-normal) vision. The study protocol was performed according to the Declaration of Helsinki and was approved by the Ethics Committee of the National Institute for Physiological Sciences (permit No. 20A063). The psychological experiments were performed with informed consent of all subjects. Informed consent included permission to disclose the subject’s initials.

## Results

### Summation rule for illusory motion

**Figure 2:**
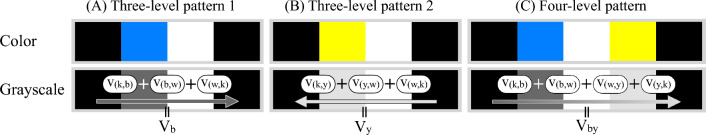
Relationship between pattern that induce illusory motion and perceived velocity. Three-level patterns consist of units arranged in two sequences (**A**,**B**): black (k), dark gray/blue (b), white (w) and black (k), light gray/yellow (y), white (w). A four-color pattern consists of units arranged in the following order (**C**): black (k), dark gray/blue (b), white (w), light gray/yellow (y). Each pattern is perceived as moving in direction indicated by a arrow at velocities Vb, Vy and Vby. Some mathematical models explain that perceived velocity is obtained by summing velocities extracted from local contrasts.

Before describing results of the experiment, the summation rule for illusory motion that was verified in this study is explained below. Figure 2 shows a schematic diagram of the summation rule. In this diagram, details of the rule are explained using three specific MIDDs: two of them are three-level MIDDs and one is a four-level MIDD. Assume that a three-level MIDD is composed of units arranged in following order from left to right: black (k), dark gray/blue (b), and white (w). And another three-level MIDD is organized as: black (k), light gray/yellow (y), white (w). Suppose four- level MIDD consists of black (k), dark gray/blue (b), white (w), and light gray/yellow (y). From these MIDDs it is assumed that we perceive velocities Vb, Vy, and Vby, respectively.

Several studies of illusory motion explain that local motion is detected from such luminance patterns^6–8,12^. In this study, it is assumed that perceived velocity is determined by summation of local velocities as follows.

$$\begin{aligned} {\text{V}}_{{\text{b}}} & = {\text{v}}_{{({\text{k}},{\text{b}})}} + {\text{v}}_{{({\text{b}},{\text{w}})}} + {\text{v}}_{{({\text{w}},{\text{k}})}} \\ {\text{V}}_{{\text{y}}} & = {\text{v}}_{{({\text{k}},{\text{y}})}} + {\text{v}}_{{({\text{y}},{\text{w}})}} + {\text{v}}_{{({\text{w}},{\text{k}})}} \\ {\text{V}}_{{\text{b,y}}} & = {\text{v}}_{{({\text{k}},{\text{b}})}} + {\text{v}}_{{({\text{b}},{\text{w}})}} + {\text{v}}_{{({\text{w}},{\text{y}})}} + {\text{v}}_{{({\text{y}},{\text{k}})}} \\ \end{aligned}$$

where v(1,2) is local velocity detected from contrast between elements 1 and 2 aligned from left to right. If positional relationship of these elements is reversed, a sign of velocity also flips. Hence, v(1,2) becomes equal to − v(2,1). From above, it is possible to write velocity perceived from four-level MIDDs Vby in terms of velocity perceived from three-level MIDDs Vb and Vy.

$$\begin{aligned} {\text{V}}_{{\text{b}}} & = {\text{v}}_{{({\text{k}},{\text{b}})}} + {\text{v}}_{{({\text{b}},{\text{w}})}} + \left( {{\text{v}}_{{({\text{w}},{\text{k}})}} - {\text{v}}_{{({\text{w}},{\text{k}})}} } \right) + {\text{v}}_{{({\text{w}},{\text{y}})}} + {\text{v}}_{{({\text{y}},{\text{k}})}} \\ & = \left( {{\text{v}}_{{({\text{k}},{\text{b}})}} + {\text{v}}_{{({\text{b}},{\text{w}})}} + {\text{v}}_{{({\text{w}},{\text{k}})}} } \right) - \left( {{\text{v}}_{{({\text{k}},{\text{y}})}} + {\text{v}}_{{({\text{y}},{\text{w}})}} + {\text{v}}_{{({\text{w}},{\text{k}})}} } \right) \\ & = {\text{V}}_{{\text{b}}} - {\text{V}}_{{\text{y}}} \\ \end{aligned}$$

Note that the sign of Vy is negative because the order of elements in three- level MIDD (k → y → w) is opposite to that in four-level MIDD (k → b → w → y). Perceived velocity is actually expected to be affected by spatial frequency of stimulus^18^. However, a number of unit repetitions is fixed for stimuli used in this experiment, so that effect can be considered negligible. In the psychophysical experiment, the summation rule were verified.

### Psychophysical experiment

**Figure 3:**
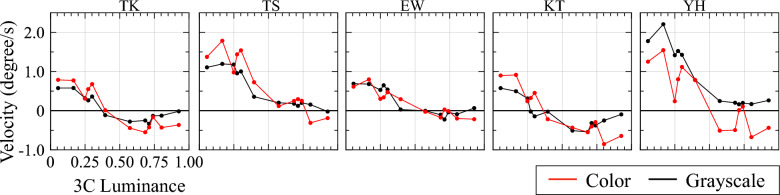
Results of the psychophysical experiment for each of the five subjects with three-level MIDDs. The vertical axis indicates perceived velocity and the horizontal axis indicates value of 3C luminance converted from hue. The red and black graphs show perceived velocity for color and grayscale stimuli, respectively. The correspondence between luminance and hue is shown in Supplementary Fig. S2.

Figure 3 shows perceived velocities of three-level MIDDs (five subjects). In addition to color MIDDs (red), grayscale MIDDs (black) were used as stimuli in the experiment. The horizontal axis indicates 3C luminance converted from hue. The luminance shown here has been normalized to white luminance after subtracting the offset caused by black luminance, as described in the Method. The correspondence between luminance and hue is shown in the Supplementary Fig. S2. Positive values on the vertical axis indicate that MIDDs were perceived to rotate clockwise. Although there are individual differences, this figure shows a clear correlation between 3C luminance and perceived velocity. The results of the analyses performed in this experiment are summarised in Table 1. The table presents mean and standard deviation of data, derived from an analysis of five subjects. Grayscale stimuli had a slightly lower correlation coefficient than color stimuli, but both visual stimuli had high correlation coefficients with luminance. When 3C luminance was varied, most subjects perceived a reversed direction of rotation, except for one subject in the case of color who did not perceive a reversed direction of rotation. Luminance levels at which this reversal occurred differed between subjects (Table 1).

**Table 1:** Analysis results on perception of illusory motion with three- or four-level MIDDs.

		Correlation coefficient between 3C/4C1 luminance and perceived velocity	Luminance when the direction of rotation is reversed	W value for Shapiro–Wilk test
Three-level MIDDs	Color	− 0.9103 ± 0.0264	0.6121 ± 0.1102 (Max: 0.7849, Min: 0.4663)	
	Gray scale	− 0.8655 ± 0.0633	0.6713 ± 0.1639 (Max: 0.8413, Min: 0.4156)	
Four-level MIDDs	Color	− 0.9190 ± 0.0144	0.1969 ± 0.0573 (Max: 0.2870, Min: 0.1152)	0.9529 ± 0.0157
	Gray scale	− 0.9137 ± 0.0347	0.3403 ± 0.0250 (Max: 0.3816, Min: 0.3122)	0.9362 ± 0.0210

**Figure 4:**
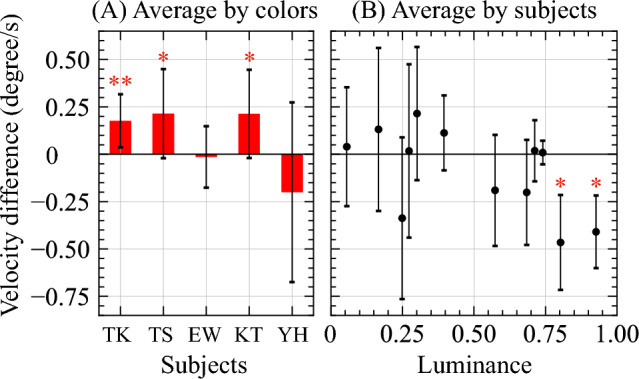
Comparion of difference of absolute values of perceived velocity for color and grayscale stimuli of three-level MIDDs. The left figure (**A**) shows difference of absolute perceived velocity averaged over all hues for each subject, where the vertical axis indicates averaged difference of absolute perceived velocity of stimulus and the horizontal axis indicates a subject. The right figure (**B**) shows difference of absolute perceived velocity averaged over all subjects for each 3C, where the horizontal axis indicates 3C luminance. Error bars indicate standard deviation. An asterisk indicates a significant difference (*p < 0.05, **p < 0.01).

Perceived velocities of color and grayscale stimuli at same luminance level were compared. Absolute value of perceived velocity of grayscale stimulus was subtracted from that of color stimulus, for each hue and subject. In this evaluation, no significant difference was found for two out of five subjects (Fig. 4A). However, when evaluating difference in perceived velocity across all subjects for each hue, we found a significant reduction only for hues with the highest and next highest luminance level, hue 60 and 90 (Fig. 4B). These results suggest that influence of color on perceived velocity may be partially limited.

**Figure 5:**
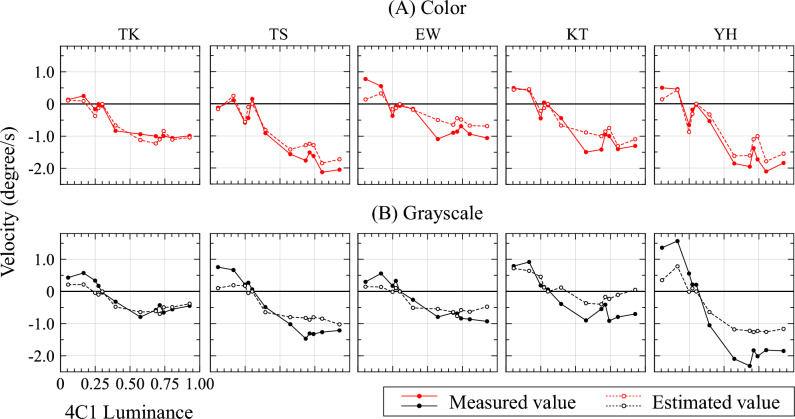
Results of the psychophysical experiment for each of the five subjects with four-level MIDDs. Dotted lines in graphs for four-level MIDDs show velocities estimated from three-level MIDDs (Fig. 3) by the summation rule. Other interpretations of this figure follow the same format as Fig. 3.

Results of the psychophysical experiment with four-level MIDDs are also shown in Fig. 5. Considering heavy load on the subjects, the number of each stimulus was limited to 12 stimuli, with hue of 4C2 fixed at 300 and hue of 4C1 varied. There appeared to be a linear correlation between 4C1 luminance and perceived velocity, similar to three-level MIDDs (Table 1). Further luminance levels for which direction of illusion was reversed were calculated, showing that the deviation of luminance levels was smaller than for three-level MIDDs (Table 1). This smaller deviation may be due to the inversion point being fixed unlike three levels: when hue of 4C1 is same as that of 4C2, stimuli are mirror-symmetric and illusory motion does not occur. Therefore, luminance level of inversion is assumed to be around 0.3011, luminance level of hue 300.

Estimated values of four-level MIDDs calculated by the summation rule based on measured values obtained for three-level MIDDs are shown as dotted lines (Fig. 5). The figure indicates that estimated and measured values for four-level MIDDs are very close. The Shapiro–Wilk test was used to evaluate normality of distribution of errors between estimated and measured values. This test is a statistical method for determining whether data are derived from a normal distribution and is characterized by its effectiveness for small sample sizes. W values obtained from test results were close to 1, indicating that errors were random and not systematically biased (Table 1). From these results, we propose that the summation rule enables the amount of the illusory motion produced by the four-level MIDD to be correctly estimated.

## Discussion

Using psychophysical methods to measure perceived velocity of illusory motion produced by MIDDs, we found that amount produced by four-level MIDDs could be predicted by simply summing amounts produced by three-level MIDDs. These results support the local velocity summation hypothesis.

When intermediate units of MIDDs were varied, there was a high correlation between luminance and perceived velocity. This result can be explained by previous studies^8,10^, where local motion information is detected by linear spatial filters and nonlinear temporal effects.

These results suggest that four-level MIDDs produce the greatest illusion when intermediate luminance is chosen to be blue (dark gray) with a luminance close to black and yellow (light gray) with a luminance close to white, similar to color composition of the Rotating snakes. Experiment results of three-level MIDDs showed that blue (hue 240) and yellow (hue 60) induced greater amounts of illusory motion in different directions. Given the summation rule, this reversal of motion direction is important: four-level MIDD can produce greater perceived velocity than three-level MIDD by making motion direction induced by combination black → 4C1 → white same as that induced by combination white → 4C2 → black. Importance of this combination in inducing illusory motion has been previously discussed^5^, but this study is the first to present its numerical evaluation.

However, when grayscale stimuli were presented with an intermediate luminance close to white, perceived velocities decreased for all subjects. This result appeared to violate linear correlation of perceived velocity to luminance. This suggests that perceived velocity was not determined by luminance alone. For example, yellow, whose luminance is close to white, can be discriminated from white, but gray with the same luminance level to yellow is difficult to discriminate from white. When grayscale MIDDs with intermediate unit close to discrimination threshold with white are presented, the previously described linearity in perceived velocity could break down. In a related study, Atala-Gérard, L. & Bach, M. observed amount of illusory motion with stimuli consisting of four grayscale elements to evaluate existing models of illusory motion^15^. When luminance levels of two intermediate units corresponding to 4C1 and 4C2 of MIDD were varied, direction of illusory motion, produced by stimuli with close to white intermediate luminance, differed from empirical predictions. They reported that linear models could not account for this result^8,10^ and showed that it could be explained by an improved form of the computational model of motion information that incorporates non-linear effects on luminance, subsequently proposed by the same group^12^.

The psychophysical experiment have confirmed that amount of illusory motion and luminance levels at which direction of illusory motion is reversed vary from person to person. Previous studies have also reported that there are such individual differences^6,8,12,18^. However, experimental results show that the summation rule is able to predict perceived velocity. Therefore, unlike the mechanism that detects motion from local information, the integration mechanism of motion perception, which is driven by additive measures, is considered to be a universal function.

### Supplementary Information

Supplementary Figures.

## Data Availability

The datasets used and/or analyzed during the current study are available from the corresponding author on reasonable request.
