# Supplementary material for: Integration of motion information in illusory motion perceived in stationary patterns
Source: Sci Rep. 2023 Nov 30;13:21107. doi: 10.1038/s41598-023-48265-4 (PMC10689723; doi:10.1038/s41598-023-48265-4)
Supplement: Supplementary file 1 — Supplementary Figures. [file 41598_2023_48265_MOESM1_ESM.docx]

Supplementary Figure S1: Representative psychometric curves of the authors (TK) for a three-level MIDD with 240 hues of 3C. To obtain an amount of illusory motion, the original image (red) and the mirror image (blue) were used in the experiment. The Kolmogorov-Smirnov test was performed to examine the goodness of fit, and the results are described.

Supplementary Figure S2: Results of the luminance calibration of the display used in the psychophysical experiment. The horizontal axis indicates the hue of the HLS color space and the vertical axis indicates the luminance. Green, blue, and red lines indicate pre-calibration, post-calibration, and theoretical luminance, respectively.
